# Prairie Dog Decline Reduces the Supply of Ecosystem Services and Leads to Desertification of Semiarid Grasslands

**DOI:** 10.1371/journal.pone.0075229

**Published:** 2013-10-09

**Authors:** Lourdes Martínez-Estévez, Patricia Balvanera, Jesús Pacheco, Gerardo Ceballos

**Affiliations:** 1 Instituto de Ecología, Universidad Nacional Autónoma de México, México, México; 2 Centro de Investigaciones en Ecosistemas, Universidad Nacional Autónoma de México, Michoacán, México; 3 Department of Biology, Stanford University, Stanford, California, United States of America; 4 Department of Ecology, Evolution, and Environmental Biology, Columbia University New York, New York, United States of America; BiK-F Biodiversity and Climate Research Center, Germany

## Abstract

Anthropogenic impacts on North American grasslands, a highly endangered ecosystem, have led to declines of prairie dogs, a keystone species, over 98% of their historical range. While impacts of this loss on maintenance of grassland biodiversity have been widely documented, much less is known about the consequences on the supply of ecosystem services. Here we assessed the effect of prairie dogs in the supply of five ecosystem services by comparing grasslands currently occupied by prairie dogs, grasslands devoid of prairie dogs, and areas that used to be occupied by prairie dogs that are currently dominated by mesquite scrub. Groundwater recharge, regulation of soil erosion, regulation of soil productive potential, soil carbon storage and forage availability were consistently quantitatively or qualitatively higher in prairie dog grasslands relative to grasslands or mesquite scrub. Our findings indicate a severe loss of ecosystem services associated to the absence of prairie dogs. These findings suggest that contrary to a much publicize perception, especially in the US, prairie dogs are fundamental in maintaining grasslands and their decline have strong negative impacts in human well – being through the loss of ecosystem services.

## Introduction

Grasslands, including pastureland, croplands sown with pasture and fodder crops, shrublands, and rangelands, covered around 3.5 billion ha in 2000, which represented 26% of the world land area and 70 percent of the world agricultural area [1]. Grasslands have been heavily impacted by agricultural and other kind of fragmentation, introduction of domestic livestock and non-native species, and the suppression of natural fire regimes, which have led to the loss of biodiversity and the increased abundance of invasive species [2]–[4]. With the rate of grassland transformations greatly exceeding their protection, most grassland biomes are classified as being critically endangered [5]. These large-scale, land use changes are reducing the capacity of these ecosystems to maintain biodiversity [6]–[8].

Direct anthropogenic impacts interact with drought into driving desertification. Desertification is defined by UN as ‘land degradation in arid, semiarid, and dry sub-humid areas resulting from various factors, including climatic variations and human activities’ [9] and is characterized by the loss of grasses and forbs and the rapid expansion of shrubs such as mesquite (*Prosopis glandulosa)* [10]. Land degradation is dependent on biophysical and socioeconomic factors, the interactions between them, and their spatial and temporal scales of influence. Some of the biophysical factors that play a key role in worldwide grasslands are the spatial distribution, soil fertility and stability, seed viability, species diversity, shrub encroachment, irrigation water, gully formation, soil erosion and composition of functional groups. On the other hand, the principal socioeconomic factors involved are labor needs, access to irrigation water, crop storage, education, access to government aid programs, selling or renting lands, conflict resolution, access to drinking water, population size, land planning, overexploitation of the resources, livestock and crops market, and migration [9].

The direct and indirect anthropogenic degradation of grasslands is likely to have paramount impacts on societies through the decline of their ability to supply key ecosystem services that are relevant to local, regional and global stakeholders. Grasslands play a key role in food production, through beef livestock production, which was 58 000 million tons in 2008 [1] as well as their transformation to agriculture, which accounts 20% of the historical area covered by world’s native grasslands [11]. Grasslands are also important for ecosystem services such as carbon storage and sequestration, estimated at approximately 34% of the global stock of carbon in terrestrial ecosystems; water infiltration and aquifer recharge; the provision of biofuels, as well as recreation [12].

Changes in the ability of grasslands to supply fundamental ecosystem services depend on complex interactions between their abiotic and biotic components, as well as their interactions with societies. Such changes are likely linked to changes in the presence of keystone species, those that have a much larger role in the structure and function of the ecosystem than expected by their abundance [13]. One of such species is the black-tailed prairie dog (*Cynomys ludovicianus*) which its geographic range once extended from southern Canada to northern Mexico; however, the species has disappeared from 98% of their original distribution range [14].

Prairie dogs are keystone species and an ecosystem engineer and are essential in maintaining grasslands at three levels: a) as ecosystem engineers they have a great impact on the physical, chemical and biological soil properties; through the construction of their burrows they aerate the soil, redistribute nutrients, add organic matter and increase the water infiltration [15], [16], b) with their foraging and burrowing activities they create unique islands of grassland habitat by maintaining a low, dense turf of forbs and grazing-tolerant grasses, contributing to the maintenance of the open grassland habitat and preventing the growth of woody plants [17]–[21], and c) they provide key habitat for many grassland animals, enhance the nutritional quality of forage, which attracts large herbivores to their colonies, and provide important prey for predators [18], [19], [22], [23]; as a component in the food chain ensure the existence of certain carnivores that depend on it, such as the black-footed ferret (*Mustela nigripes*). In addition, their burrows are important refuges for species of amphibians, reptiles, birds and other mammals [15], [16], [24]. The negative impacts of prairie dogs extirpation are, among many others, the regional and local biodiversity loss [24], [25], the increased seed depredation [26], and the promotion, establishment and persistence of invasive shrubs [10].

One of the largest black-tailed prairie dog complex have persisted in the grasslands of Janos biosphere reserve, northwestern Chihuahua, Mexico, which is one the most important sites for conservation of the grasslands of North America [27]–[29]. These colonies survived because until the late 1990’s the region lacked electricity and the local communities dominated by fundamental Mennonite communities refused to use modern technological methods for agriculture [30], [31]. Unfortunately, that changed and grassland degradation in the Janos region has been accentuated in recent years, due to the acceptance of modern agricultural methods that have lead to encroachment by agriculture and livestock overgrazing, the illegal exploitation of aquifers, suppression of fire regimes, and wildlife eradication [2]. Despite these anthropogenic pressures on the ecosystem, there are still large areas with prairie dogs colonies that have become the most important element for the conservation of this ecosystem and its species. The large extension of the prairie dog complex in the Janos region has provide us with a unique opportunity to evaluate the role of prairie dogs in the structure, function, and conservation of semiarid grasslands [2], [15], [27]. In this work we assess the role played by the black – tailed prairie dog in the supply of the key ecosystem services from grasslands.

We carried out our study in the Janos Biosphere Reserve because it supports a large prairie dog population, but also harbors areas in which the species has been recently extirpated giving rise to pastures and to areas dominated by mesquite. We investigate the effect of prairie dogs on five ecosystem services, including groundwater recharge, regulation of soil erosion, regulation of soil productive potential, soil carbon storage and forage availability. Specifically, we address the following questions: 1) What is the scope of the ecosystem services in the prairie dog grasslands? 2) How does supply of such ecosystem services change when prairie dogs have become locally extinct and grasslands or mesquite scrub have developed? 3) What are the conservation implications of the presence (or absence) of prairie dogs and ecosystem services to human well being?

## Methods

### Study Site

**Figure 1:**
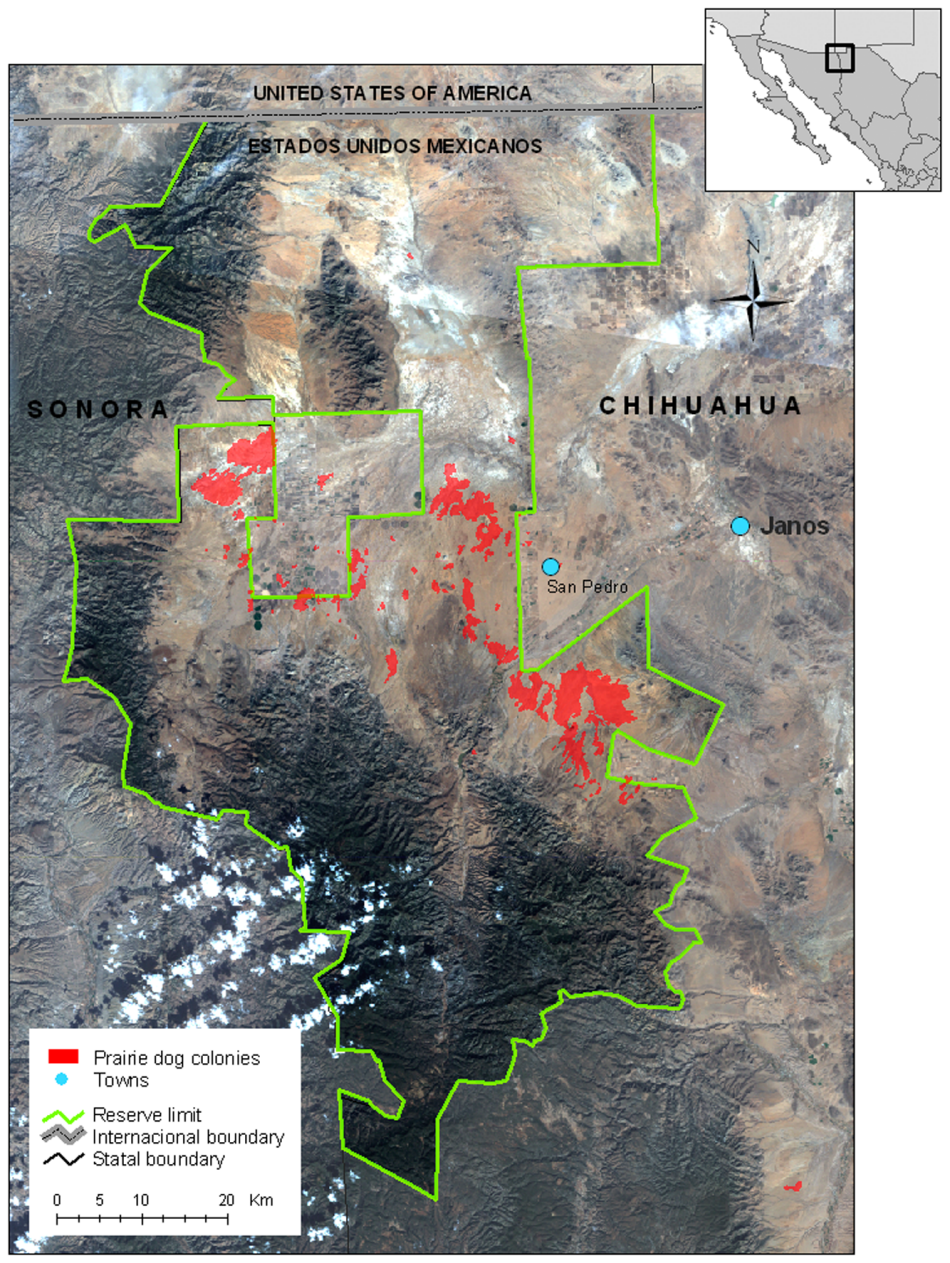
The study area was located in the Janos Biosphere Reserve, in northern Chihuahua, close to the Mexico – US (New Mexico) international border.

The study was conducted in the black-tailed prairie dog complex located in the Janos Municipality in northwestern portion of the Mexican state of Chihuahua. The arid grasslands supporting these colonies extend northeast of the Sierra Madre Occidental to 75 km south of the U.S.-Mexico border (30° 50′N, 108° 24′W; Figure 1). The elevation is 1400 m above the sea level with the topography characterized by expansive arid plains with slopes of less than 5% and bounded by ridges and hills with 12 to 30% slopes [32]. According to the Köppen climate classification, modified by Garcia [33] the climate is arid (BSokw (e′). The temperatures range from 15°C in winter to 50°C in summer, with a mean annual temperature of 15.7°C [33]. The mean annual precipitation for this region is 307 mm [33] and displays bi-modal patterns, raining during the winter and summer growing season (July through September). The area receives occasional snowfall during the winter, with the spring (March–June) being characteristically dry with frequent high winds from the southwest. Humidity is low during most of the year [33].

The vegetation is dominated by a mixture of forbs and annual grasses with patches of perennial grasses in grasslands with and without prairie dogs, and by mesquite shrubs in shrubs sites. Dominant species across the region include *Aristida adscensionis*, *Aristida divaricata, Bouteloua aristidoides*, *Bouteloua gracilis*, *Bouteloua eriopoda*, *Prosopis glandulosa*, *Festuca imbricata*, *Pleuraphis mutica*, *Opuntia imbricata*, *Yucca canerosana* and *Ephedra trifurca* [32].

#### Characterization of the study area

Sites with similar soil and landform features but contrasting land cover resulting from prairie dog extirpation were identified, by analyzing topographic (Carta topografica Nacozari H12-6 1:250,000) and soil maps (Carta edafologica Nacozari H12-6 1:250,000) of the area. To assess the physical soil features of selected sites 36 soil drillings and12 pits (2 m depth, 3 m length) were made. Soil samples were taken in each horizon for laboratory analyses. The dominant landform in the study area is the arid alluvial fans with sporadic runoff and little deposition. The dominant soil in the region, according to the classification of the Soil Survey Staff [34], is the Mollisol represented by two main groups: Argiustols and Haplustols.

#### Treatments and local plots

Three contrasting conditions (hereinafter called “treatments”) were identified along a successional continuum associated to prairie dog extirpation: grasslands currently occupied by prairie dog (prairie dog grasslands); grasslands occupied by prairie dogs 10 years ago (grassland), and grasslands previously occupied by prairie dogs (roughly 10 years ago) and currently dominated by mesquite (mesquite). Six 50×50 m plots were established for each treatment within the continuum for a total of 18 plots; all of them were in mollisols at the lower margins of the alluvial fan and with a minimum distance between them of one kilometer to ensure the plots were independent. Within each plot, three 50X1 mts transects length were systematically positioned at 16 m intervals. Measurements were collected during the spring of 2010.

### Evaluation of Ecosystem Services

The ecosystem or environmental services evaluated were groundwater recharge, regulation of soil erosion, regulation of soil productive potential, soil carbon storage and forage availability. We evaluated them as follows:

#### Groundwater recharge

Water infiltration was defined as the rate at which water enters the soil and is relevant to ground-water recharge. Ground-water is the main source of water for agricultural activities in the area [2]. Water infiltration was measured using the single-ring infiltrometer method [35]. A fixed volume of water (50 mm) is poured on a soil area under saturated conditions and the time needed for the total amount of water to enter the soil is recorded. The infiltrometers were systematically positioned every 10 m along the 3 transects within each plot, in a total of 15 points of measurement per plot. In the case of the mesquite treatment and according to the transects, the infiltrometers were randomly placed both under shrubs and interspaces between mesquites.

#### Regulation of soil erosion

Regulation of soil erosion was defined as the absence of evidences of soil erosion, and is a key service to owners of the plots. Soil erosion was assessed based on two evaluations:

- Soil erosion indicators

- The presence or absence and intensity of nine qualitative soil erosion indicators were assessed following the Pellant et al. protocol [36]. The indicators included the presence of: a) rills, b) water flow patterns, c) pedestals and/or terracettes, d) bare ground, e) gullies, f) wind depositional areas, g) litter movement, h) soil surface resistance to erosion, and i) soil surface loss or degradation. For each of the indicators a rank value was provided based on their qualitative intensity: none to slight (ranking value = 1), slight to moderate (2), moderate (3), moderate to extreme (4), extreme to total (5). A synthetic index was built with the sum of all ranking values for each of the nine indicators. Lower values indicated greater soil stability.

- Soil protection through land cover

- Four classes of land cover were evaluated: plant, litter, rocks, and bare ground using a 50 m line-point-intercept [35] with point observations obtained every 50 cm, for a total of 100 readings per line and 300 readings per plot. Plant life-forms cover was also evaluated; the frequency of observations for each species was recorded with each one assigned to a specific life-form group (annual herbs, perennial herbs, annual grasses, perennial grasses and shrubs). The amount of grasses and herbs is a measure of habitat condition, with the greater the abundance of grasses, the better the habitat quality.

#### Regulation of soil productive potential

Regulation of soil productive potential was defined as the inverse of soil compaction; this is a key service to plot owners and to the inhabitants of the regions as it is associated to the regulation of soil productive potential, in particular to the development of radicular systems [37], as well as to the role played by soils in the regulation of runoff and infiltration [38].

Regulation of soil structure was measured using a static cone penetrometer Rimik CP20. Ten lectures, randomly established per plot, were taken to measure the resistance to penetration in KPa and to a maximum depth of 60 cm. In the case of the mesquite treatment, five lectures were taken under the shrubs and the other five in the interspaces. The higher the penetration the smaller the compactation.

#### Soil carbon storage

Soil carbon storage was defined as soil carbon content per unit volume of soils; this is a key service to the global community, given that grasslands are an important reservoir of carbon according to their worldwide extension.

Soil carbon content was assessed in three soil pits (2 m depth and 3 m length) per treatment. Soil samples (150 gr.) from the different horizons of each profile were taken and then analyzed with the Elemental Analyzer CHNS/O Perkin Elmer 2400 series II. An edaphoecological evaluation was conducted to obtain additional information of soils physical features to calculate carbon sequestration [39].

#### Forage availability

Forage was defined as the amount of forbs and grass biomass per unit area; the amount of total potential biomass to be consumed by cattle is a key ecosystem service to local ranchers.

To determine the amount of forbs and grass biomass for each class, six ¼ m^2^ (50×50 cm) quadrants were clipped by species along each transect at a height of 1 cm of above the soil surface, placed in labeled paper bags, dried in a forced air oven at a temperature of 58°C for 48 hours, air equilibrated, then weighed. Dr. Toutcha Lebgue Keleng, curator of the University of Chihuahua (UACH) herbarium, verified plant identification.

All the permissions and permits required for the fieldwork were requested and authorized by the private landowners, Janos municipality, communal land owners, and by the administration of The Nature Conservancy Ecological Reserve “El Uno”.

### Data Analysis

The assumptions of data normality were assessed using the Shapiro-Wilks modified test. Data were analyzed using InfoStat program [40]. To determine differences in water infiltration and soil productive potential between treatments, a Kruskal-Wallis test was used followed by post-hoc multiple comparison test (Dunn’s method) [41]. For differences in soil erosion between treatments a contingency table was used followed by a correspondence analysis. For soil carbon storage Friedman two way non parametric test was used to determine differences between treatments and also a Kruskal-Wallis test was used to determine differences in soil carbon storage between horizons. To determine the differences between treatments in terms of soil protection trough land cover an ANOVA test was performed followed by Tukey post-hoc multiple comparison test; also, Kruskal-Wallis test was performed to test differences according to plant life-forms cover followed by post-hoc multiple comparison test [41]. Finally, for test differences in forage biomass between treatments an ANOVA followed by Tukey post-hoc multiple comparison tests were performed.

## Results

**Figure 2:**
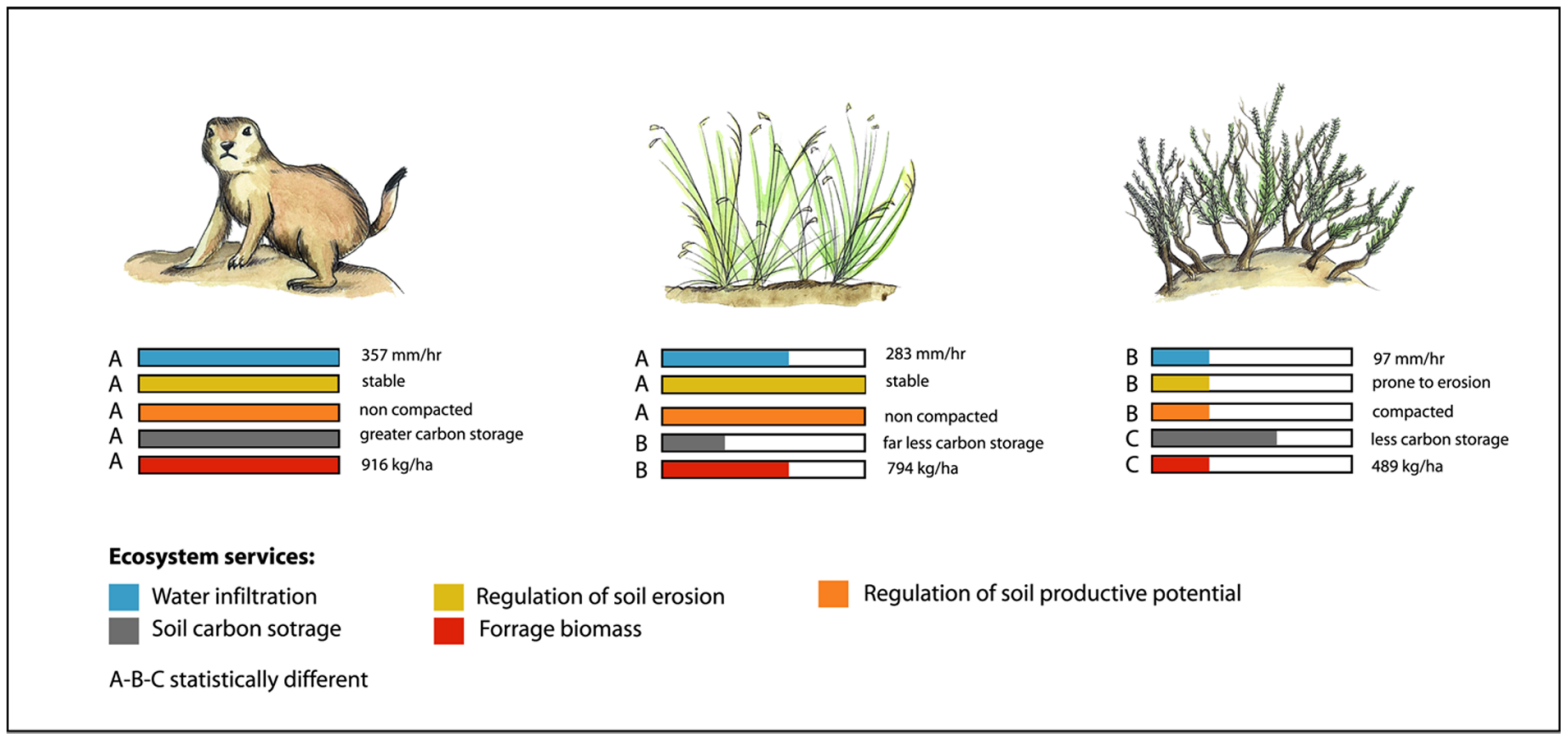
Ecosystem services were evaluated in grasslands with prairie dogs, grasslands and mesquite scrubs that have lost prairie dog colonies in the last 10 years, in the Janos region, Chihuahua, Mexico. Our results clearly show the positive effect of prairie dogs on the provision of these ecosystem services. Color bars indicate the relative magnitude of each of the services in the different treatments with respect to grasslands with prairie dogs.

In summary, our results indicate that black- tailed prairie dogs played an important role in providing ecosystem services. Grasslands with populations of prairie dogs (prairie dog grasslands hereafter) showed a higher supply of all the ecosystem services assessed here relative to areas devoid of prairie dogs that are now dominated by grasses (grasslands hereafter) or scrublands dominated by mesquite (mesquite hereafter). Prairie dogs had a very strong positive effect on the supply of groundwater recharge, regulation of soil erosion, regulation of soil productive potential, soil carbon storage, and forage (Figure 2).

### Groundwater Recharge

**Figure 3:**
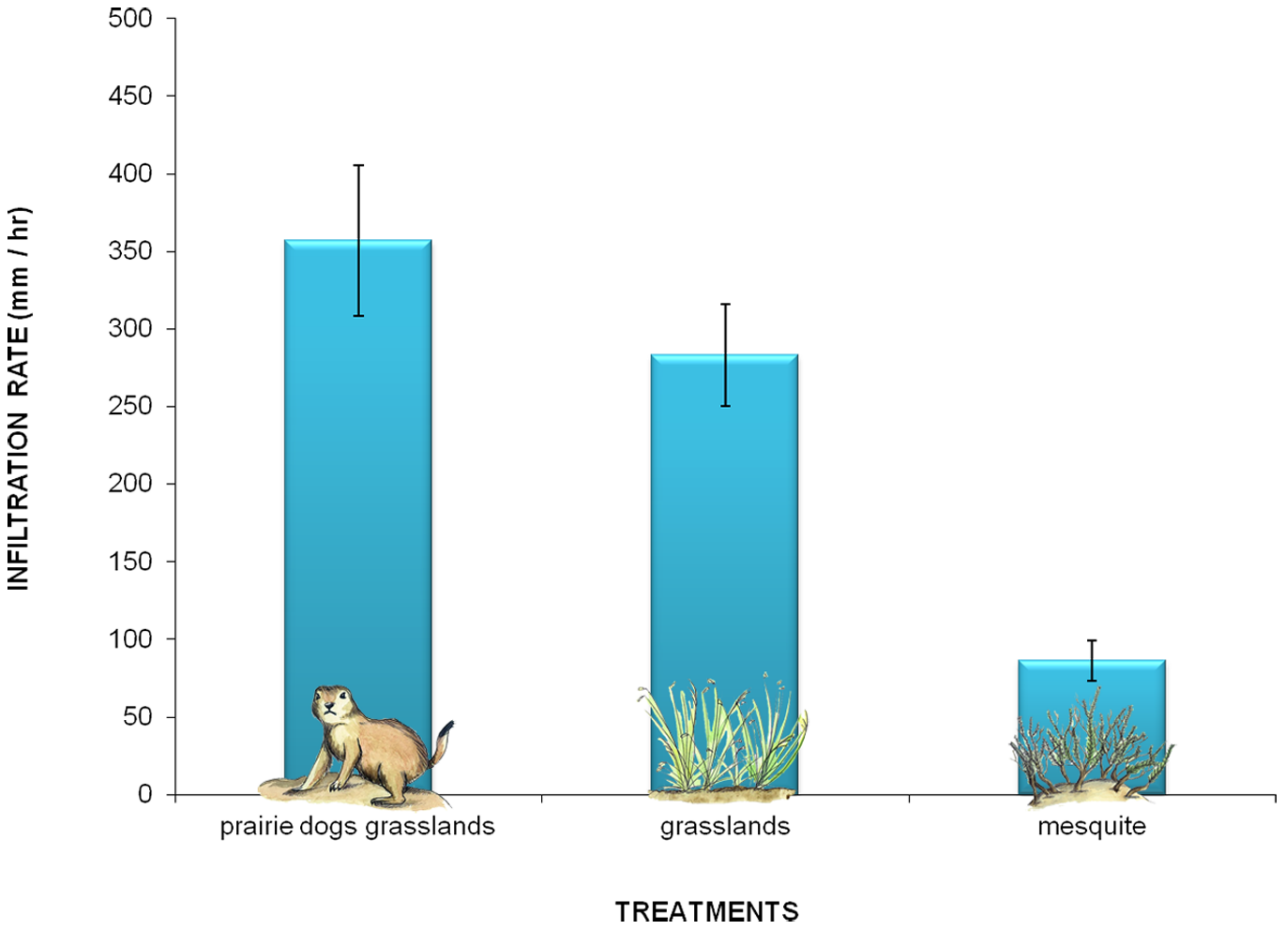
Groundwater recharge variation among treatments in the Janos region, Chihuahua, Mexico. Related to the foraging and burrowing activities of prairie dogs, among other physical and biological factors, the supply of this service was statistically higher in grasslands with prairie dogs than in grasslands and mesquite.

Prairie dogs were associated to habitats with higher water infiltration. Water infiltration rates were highest in prairie dog grasslands (357±288.45 mm/hour), followed by those of grasslands (283±194.36 mm/hour), and mesquite (97±77.82 mm/hour; Kruskal-Wallis H = 108.48 d.f. = 2 P<0.0001; Figure 3). Mesquite differed significantly from prairie dog grasslands and grasslands but these two did not differ between each other (multiple comparison: D_i, j_ = 9.790, D_i, j_ = 7.973, D_i, j_ = 1.817).

### Regulation of Soil Erosion

#### a) Soil erosion indicators

Soil stability and erosion indicators, such as bare ground, litter movement, and water flow patterns, showed that mesquite scrubland soils were the least stable and more prone to soil erosion (overall ranking value = 55 points) than prairie dog grasslands (overall ranking value = 22) and grasslands (overall ranking value = 14; Table S1). The differences in erosion indicators (e.g. bare ground) were statistically significant among treatments (X^2^ = 25.14 P = 0.0015). A correspondence analysis also showed that grasslands with and without prairie dogs were classified in the slight to moderate rating category, indicating that those plant communities are better in preventing soil erosion and had greater soil stability than mesquite scrubs, which were classified in the extreme categories (Figure S1).

#### b) Soil protection through land cover

**Figure 4:**
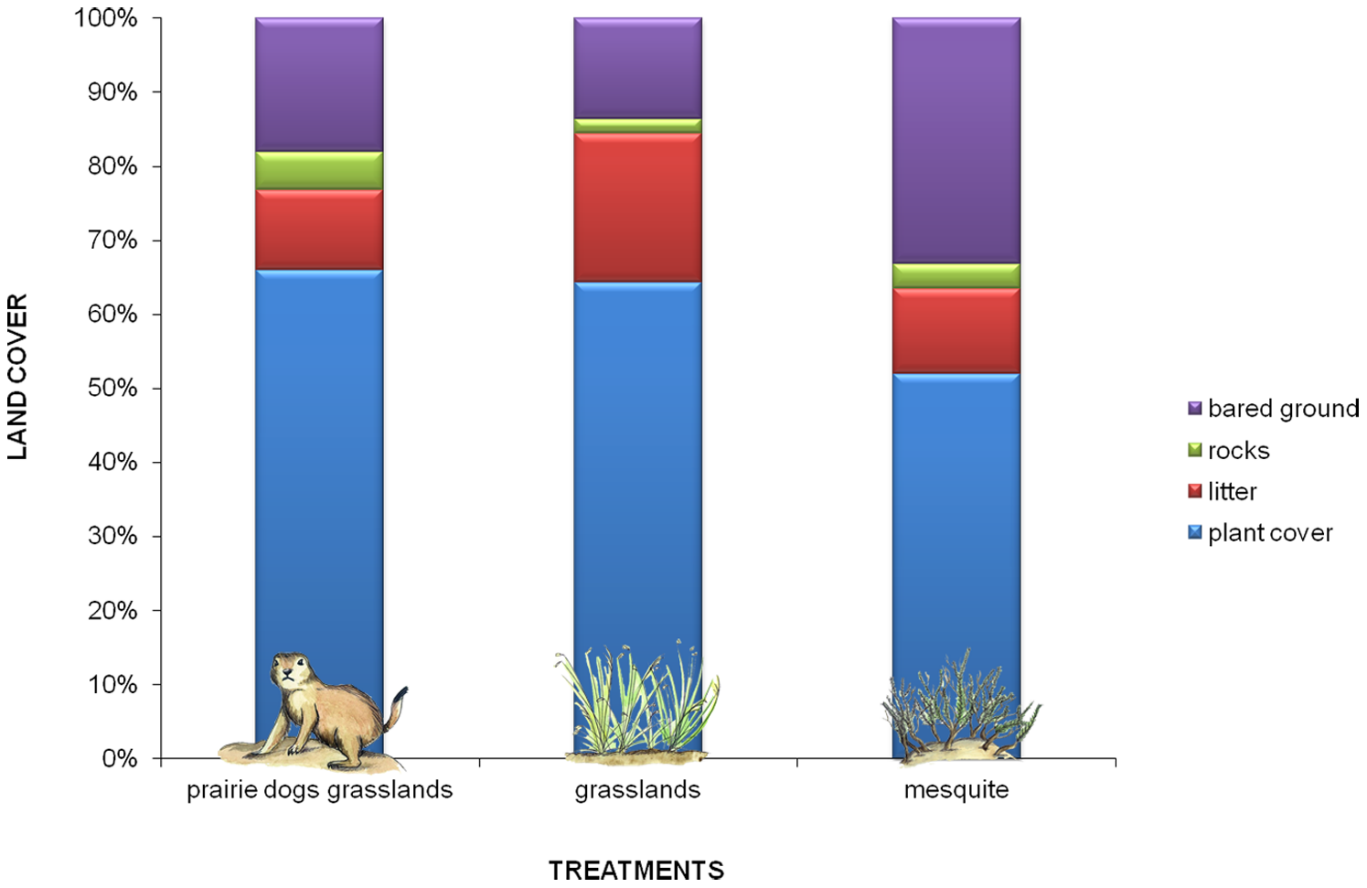
Variation in the regulation of soil erosion among treatments in the Janos region, Chihuahua, Mexico. Soils in prairie dog grasslands were less prone to erosion, because they showed more than 60% of plant cover, while mesquite soils had more than 30% bared and prone to erosion.

Plant cover within the prairie dog grasslands (66%) and grasslands (64%) tended to be greater than mesquite scrubs (51%); however, these values were not significantly different (ANOVA F = 1.51 d.f. = 2 P = 0.2534; Figure 4). A related metric (percentage of bare ground) was significantly different between grasslands (13%) and the other treatments, prairie dog grasslands (18%) and mesquite scrub (33%), but the latter were not statistically different (ANOVA F = 4.67 d.f. = 2 P = 0.0265; multiple comparison: Tukey MSD = 47.51196; Figure 4). Percentage cover by class of plant life-forms (annual forbs, perennial forbs, shrubs, annual grasses and perennial grasses) varied across the treatments (Kruskal-Wallis H = 18.54 d.f. = 2 P<0.0001). Annual forbs was the most abundant plant-life form (multiple comparison: P = 0.03), being statistically different within grasslands (80.2%; Kruskal –Wallis H = 7.21 d.f. = 4 P = 0.0154; multiple comparison: P = 0.03) and prairie dog grasslands (85.5%; Kruskal –Wallis H = 21.76 d.f. = 4 P<0.0001; multiple comparison: P = 0.002) but not within mesquite treatment (60.4%). Perennial grass cover was minimal in all treatments with average estimates of 1% of total plant cover for prairie dog grasslands and grasslands, and 7% of the mesquite scrubs. The low values for perennial grasses may be due to a 15-year drought and overgrazing [42]. Finally, shrub cover was much greater in mesquite treatment (15%) compared to the prairie dog grasslands and grasslands where prairie dogs were absent (Figure S2).

### Regulation of Soil Productive Potential

**Figure 5:**
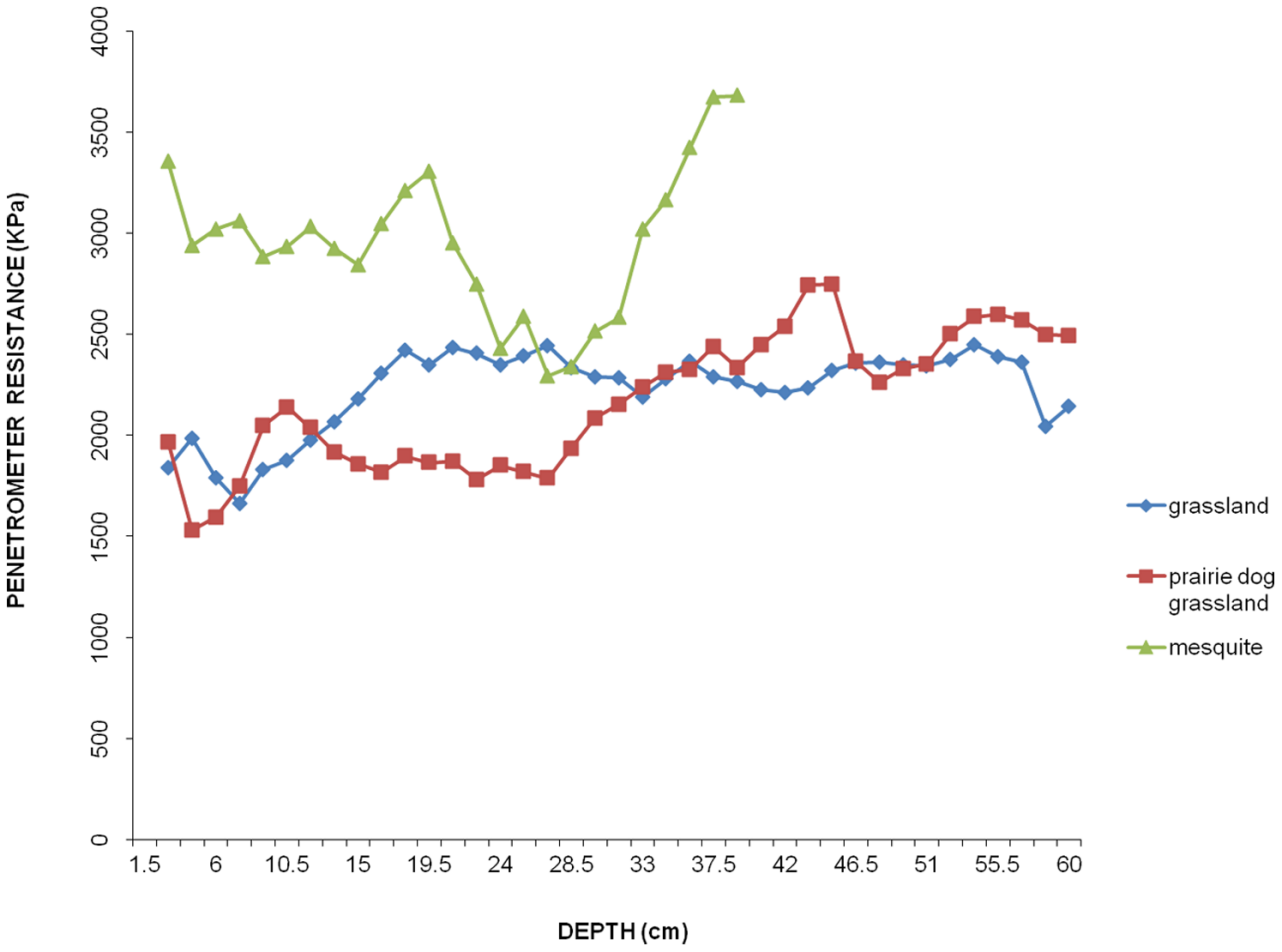
Regulation of soil productive potential service among treatments in the Janos region, Chihuahua, Mexico. Penetration resistance was higher in mesquite scrubs sites when compared with prairie dog grasslands and grasslands, indicating that soils are more compacted making more difficult the establishment of herbs and grasses.

Soil productive potential was negatively affected in mesquite scrubland, which had soils that were more compacted up to a depth of 60 cm compared to grasslands and prairie dog grasslands. These two treatments were statistically different from the mesquite treatment (Kruskal-Wallis H = 68.35 d.f. = 2 P<0.0001; multiple comparison: P<0.0001; Figure 5).

### Soil Carbon Storage

Carbon storage is related to soil productivity and carbon emissions. In our study, soil carbon storage was greater in prairie dog grasslands followed by mesquite scrubs and then by grasslands (Kruskal-Wallis H = 6.26 d.f. = 2 P = 0.0436). Within treatments, carbon concentrations were statistically larger in deeper horizons in prairie dog grasslands (Kruskal-Wallis H = 9.83 P = 0.0433). However mesquite scrub and grasslands had similar values in all horizons (Kruskal-Wallis H = 3.40 P = 0.1394 H = 1.56 P = 0.6676, respectively; Table S2). We used a two-way ANOVA to evaluate the interactions between treatments and depth effect and we found no differences between them (Friedman T^2^ = 1.34 P = 0.2855). When the prairie dog grasslands are converted into grasslands carbon storage is reduced. Carbon storage in mesquite scrubland was slightly smaller than prairie dog grasslands, indicating that mesquite scrublands may also mitigate carbon emission but this service is negatively affected because of the presence of empty soil interspaces that have minimum carbon storage.

### Forage Availability

**Figure 6:**
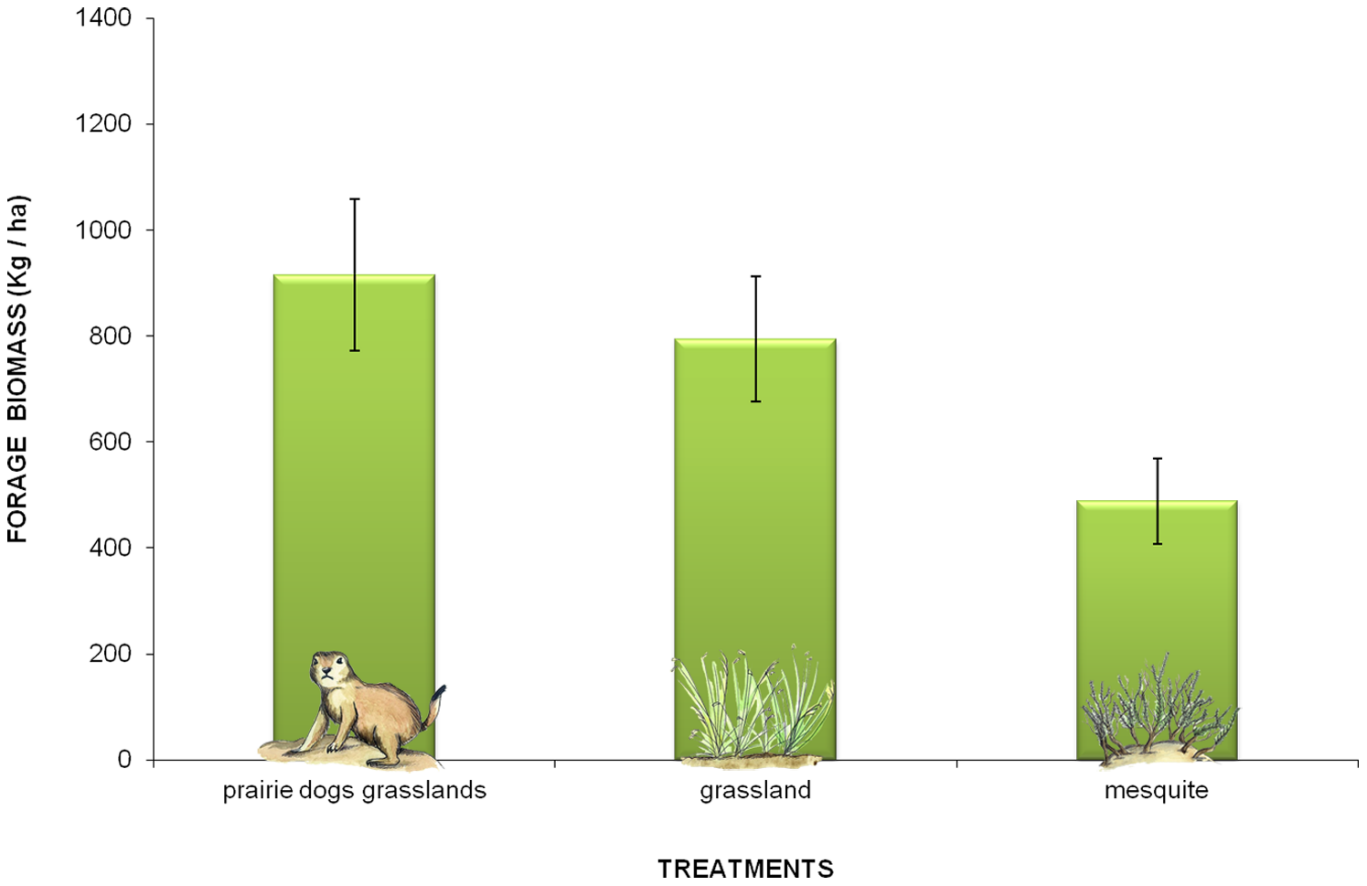
Forage availability among treatments in the Janos region, Chihuahua, Mexico. Contrary to a common lay and scientific assumption, forage availability was statistically higher in grasslands with prairie dogs than in grasslands and mesquite scrubs.

Standing biomass, available to cattle as forage, was greater in prairie dog grasslands (916±932.60 kg/ha) when compared with grasslands (794±766.04 kg/ha) and mesquite scrubs (489±524.45 kg/ha) (ANOVA F = 6.80 d.f. = 2 P = 0.0013; multiple comparison: Tukey MSD = 254.56; Figure 6).

## Discussion

Our results clearly demonstrate a strong link between prairie dogs and the provision of ecosystem services. In general, prairie dogs influenced the structure and function of the plant communities as an outcome of their feeding and burrowing activities and such impact positively affects the provision of a wide range of ecosystem services linked to both abiotic and biotic characteristics. Some of these effects have been previously reported for a variety of habitats within the black-tailed prairie dogs’ historic geographic range e.g. [13], [24], [25]. However, this is the first study that explicitly quantifies the effect of prairie dogs on specific ecosystem services. Prairie dogs support key regional socio-economic activities and are therefore directly related to human well-being. For instance, livestock benefits from cattle foraging on the edges of prairie dog colonies because of forage quality (Sierra, unpublished data). Thus, our study has strong implications for the conservation of the prairie dog in the grasslands of northern Mexico and adjacent southwestern USA.

The distribution of black-tailed prairie dogs has been greatly reduced because of habitat transformation into crops and by direct extermination by shooting and poisoning [17], [43]. Black – tailed prairie dogs can reach high densities and are believed to compete with cattle for forage, but there is increasing evidence that under suitable management both species can benefit. A similar case occurred with prairie dogs and bison (*Bison bison*) years ago. Bison prefers feeding on the edges of the colonies where forage quality was better, and at the same time their movements and foraging, allowed prairie dogs to colonize other grassland areas [44; Sierra, unpublished data].

The ecosystem services related to prairie dog presence can be attributed to higher primary productivity that benefit cattle and improved soil stability, with the attendant benefits of lower risk of erosion, flooding and desertification [see also 2, 20]. Recent studies have shown similar beneficial relationships by other small mammals, such as marmots and pikas, which act as keystone species in the maintenance of grasslands in different regions of the northern hemisphere [16], [21], [43].

The provision of ecosystem services by black-tailed prairie dogs is through complex interactions that we are only beginning to unravel. Soils within prairie dog grasslands are less compacted than soils within sites dominated by mesquite; they have more pore spaces needed for aeration and water movement within the soil profile, and have better conditions that improve their stability. These soil conditions increase water infiltration, a critical process in these semiarid grasslands where annual rainfall is less than 600 mm and ecological processes are governed by pulses of water [45]. The span of these pulsed inputs of water (summer rains are shorter than winter rains) affects nutrient cycles because the capacity of microbiota to decompose the organic matter and the ability of plants to use the nutrients depends on water availability [46]. Although nutrient turnover is a complex process depending on factors such as soil texture, vegetation patch structure, soil biota, and climate, the synchrony between nutrient availability and the capacity of plants to use available nutrients may be enhanced by prairie dog activities that increase infiltration.

The increase in soil water and prairie dog grazing improves forage production for cattle, which was demonstrated by our results on plant cover, and was consistent with data reported in the literature e.g. [47]. Aboveground plant production increases with moderate grazing because it accelerates the rates of mineralization of inorganic nutrients [47]. In addition, grazer like prairie dogs remove senescent leaves that decrease light and soil water for younger and more active tissues that also have greater nutrient concentrations for herbivores. Then herbivores can improve harvesting efficiency in terms of available nutrients per bite, and bites per minute, and increase the diversity and productivity in the grasslands [17], [48]. In contrast, mesquite encroachment affects water infiltration and drainage, and limits the soil’s capacity to support the grasses and forbs needed by native herbivores and economically important livestock species. Soils in mesquite scrubs are compacted and have crusts on the surface; such features are associated with land degradation, reduced yield of useable forage and increasing surface water runoff and flooding [10], [49]. Increased provisions of forage benefit local people by reducing cost of supplemental feeds and for improving the vigor of forage species. In the Janos region, 78% of the land is used for grazing.

From a global perspective, the maintenance of prairie dog grasslands mitigates impending climate change by storing atmospheric carbon. Semiarid grasslands, which cover 9 million km^2^ in temperate regions, store from 10 and up to 30% of world soil carbon [50]. Grasslands converted to agricultural practices typically release soil carbon to the atmosphere and continued tillage of grasslands for intensive agriculture can compound the existing problem of atmospheric carbon pollution.

The loss of the black-tailed prairie dog resulting in the transition to a desertified mesquite scrub, negatively impact the landscape’s ability to provide ecosystem services essential for local and regional natural communities and human well-being. In the Janos region, over the last 10 years, grasslands have lost more than 47,000 ha of ground cover, bare ground with no plant cover has increased from 6,645 ha to 152,123 ha, 73% of the 55,000 ha of prairie dog colonies have been lost and mesquite and ephedra (*Ephedra trifurca*) shrubs have encroached grass areas [2], [51].

The fact that some of the services evaluated did not show significant differences between treatments is likely explained due to the low densities of prairie dogs. The region has experienced an intense drought period (1995-to date), that accelerated land degradation processes and therefore the decline of prairie dogs. These conditions have diminished the role of prairie dogs in shaping the structure and function of their environment. Nevertheless, the differences in our data suggest that prairie dog populations within a given density are the main driver in the maintenance and improvement of the ecosystem services evaluated. Is clear that is necessary to preserve prairie dog populations ecologically functional for maintaining these services on the long term.

Our conservation work on the Janos grasslands focuses on developing strategies with local and state governments to maintain and restore grasslands, their biodiversity, and the ecosystem services they provide. Prairie dog management to prevent desertification resulting from mesquite replacement of native grasslands is cost-effective, relative to the high cost (US $58 per ha) for the removal of mesquite and grassland restoration [52]. A major challenge for the long-term conservation of the prairie dogs is the implementation of adequate management practices with cattle grazing and agriculture. We are developing novel techniques to couple the management of prairie dogs and cattle to maintain the grasslands, the intensive agriculture, and to restore mesquite scrubland in to grasslands.

Today’s environmental challenges require an understanding of the processes of ecosystems and wildlife populations and an ability to integrate scientific research into decision-making. Ecologists and conservation biologists must adopt this approach if we are to preserve the world’s biodiversity. To this end, the Janos Biosphere Reserve in Mexico is a global modal of commitment to the conservation of grasslands and their biodiversity.

## Supporting Information

Figure S1 **Regulation of soil erosion service.** Soil erosion indicators demonstrated that mesquite soils are more prone to erosion (extreme categories) when compared with prairie dog grasslands and grasslands (slight categories).
(TIF)

Figure S2 **Regulation of soil erosion service.** Forbs are the predominant plant life-form in prairie dog grasslands, grasslands and mesquite scrubs, being prairie dog grasslands the treatment with more percentage of them.
(TIF)

Table S1 **Presence and intensity of the qualitative indicators of soil erosion.**
(DOC)

Table S2 **Carbon sequestration in grasslands, prairie dog grasslands and mesquite scrubs.**
(DOC)
