# Supplementary figures and images for: Prairie Dog Decline Reduces the Supply of Ecosystem Services and Leads to Desertification of Semiarid Grasslands
Source: PLoS One. 2013 Oct 9;8(10):e75229. doi: 10.1371/journal.pone.0075229 (PMC3793983; doi:10.1371/journal.pone.0075229)

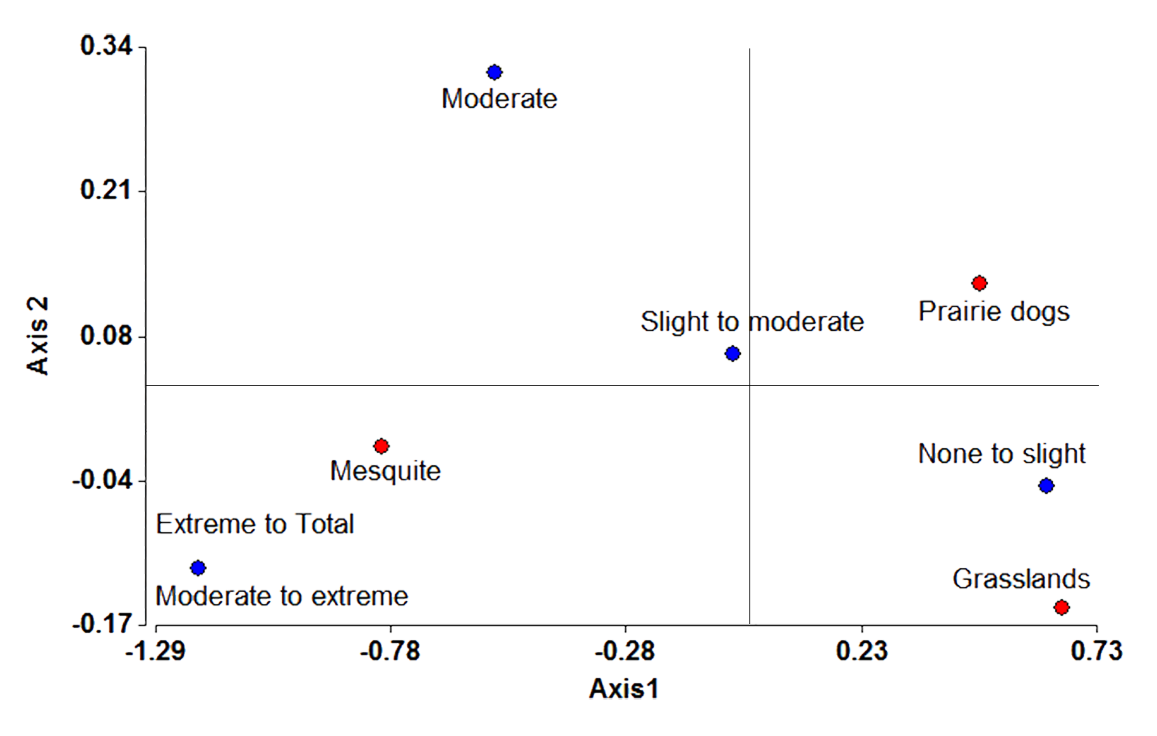

Supplement: Figure S1 — Regulation of soil erosion service. Soil erosion indicators demonstrated that mesquite soils are more prone to erosion (extreme categories) when compared with prairie dog grasslands and grasslands (slight categories). (TIF) [file pone.0075229.s001.tif]

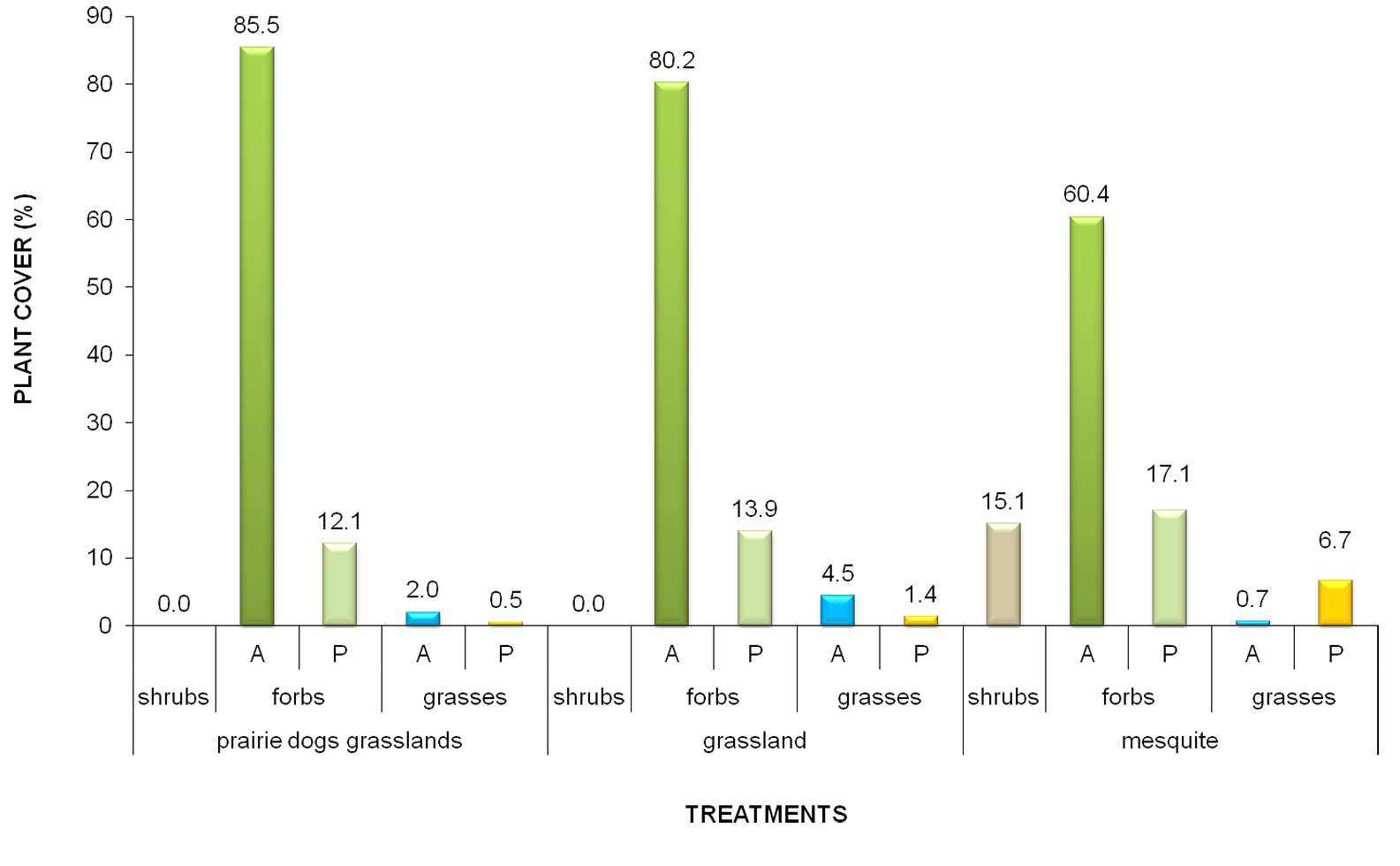

Supplement: Figure S2 — Regulation of soil erosion service. Forbs are the predominant plant life-form in prairie dog grasslands, grasslands and mesquite scrubs, being prairie dog grasslands the treatment with more percentage of them. (TIF) [file pone.0075229.s002.tif]
