# Supplementary material for: Prairie Dog Decline Reduces the Supply of Ecosystem Services and Leads to Desertification of Semiarid Grasslands
Source: PLoS One. 2013 Oct 9;8(10):e75229. doi: 10.1371/journal.pone.0075229 (PMC3793983; doi:10.1371/journal.pone.0075229)
Supplement: Table S1 — Presence and intensity of the qualitative indicators of soil erosion. (DOC) [file pone.0075229.s003.doc]

| **Indicator of**  **soil erosion** | **Mesquite** | **Prairie dogs** | **Grasslands** |
| --- | --- | --- | --- |
| **Rills** | none to slight | none to slight | none to slight |
| **Water flow patterns** | slight to moderate  moderate to extreme | none to slight | slight to moderate |
| **Pedestals and/or terracettes** | moderate to extreme | none to slight | none to slight  slight to moderate |
| **Bared ground** | slight to moderate moderate  extreme to total | none to slight  slight to moderate | none to slight |
| **Gullies** | none to slight | none to slight | none to slight |
| **Wind Scoured, Blowout, and/or Depositional Areas** | slight to moderate  moderate to extreme  extreme to total | none to slight  slight to moderate | none to slight  slight to moderate |
| **Litter movement** | slight to moderate moderate to extreme  extreme to total | none to slight slight to moderate  moderate | none to slight |
| **Soil surface resistance to erosion** | slight to moderate  moderate to extreme | none to slight  slight to moderate | none to slight |
| **Soil surface loss or degradation** | slight to moderate  moderate | none to slight  slight to moderate | none to slight |
| **Total ranking value** | 55 | 22 | 14 |

Ranking values: none to slight (1), slight to moderate (2), moderate (3), moderate to extreme (4) and extreme to total (5).
