# Supplementary material for: Prairie Dog Decline Reduces the Supply of Ecosystem Services and Leads to Desertification of Semiarid Grasslands
Source: PLoS One. 2013 Oct 9;8(10):e75229. doi: 10.1371/journal.pone.0075229 (PMC3793983; doi:10.1371/journal.pone.0075229)
Supplement: Table S2 — Carbon sequestration in grasslands, prairie dog grasslands and mesquite scrubs. (DOC) [file pone.0075229.s004.doc]

|  | **Horizons** | **Apparent density (AD)** | **pH** | **OM**  **(%)** | **Depth (dm)** | **Organic carbon**  **(%)** | **Carbon sequestration OC * AD * depth (Kg/m^2^)** |
| --- | --- | --- | --- | --- | --- | --- | --- |
| **Grassland** | h1 | 1.2 | 7 | 1.2 | 1 | 0.62 | 0.74 |
|  | h2 | 1.4 | 7 | 0.9 | 1 | 0.48 | 0.67 |
|  | h3 | 1.7 | 7 | 0.8 | 1 | 0.47 | 0.80 |
|  | h4 | 1.4 | 8 | 0.7 | 6 | 0.35 | 2.94 |
| **Grassland** | h1 | 1.36 | 7 | 1.04 | 1 | 0.52 | 0.71 |
|  | h2 | 1.68 | 7 | 0.7 | 3 | 0.37 | 1.86 |
|  | h3 | 1.67 | 7 | 0.7 | 8 | 0.35 | 4.68 |
|  | h4 | 1.43 | 7 | 0.1 | 8 | 0.07 | 0.80 |
| **Grassland** | h1 | 1.45 | 7 | 1.08 | 2 | 0.54 | 1.57 |
|  | h2 | 1.17 | 7 | 1.28 | 2.5 | 0.64 | 1.87 |
|  | h3 | 1.36 | 7 | 0.8 | 2 | 0.41 | 1.12 |
|  | h4 | 0.95 | 8 | 0.16 | 14 | 0.08 | 1.06 |
| **Prairie dogs grassland** | h1 | 1.36 | 7 | 1.04 | 1 | 0.52 | 0.71 |
|  | h2 | 1.71 | 7 | 1.52 | 2 | 0.76 | 2.60 |
|  | h3 | 1.43 | 7 | 1.72 | 3 | 0.86 | 3.69 |
|  | h4 | 1.47 | 8 | 5.64 | 5 | 2.82 | 20.73 |
|  | h5 | 1.47 | 8 | 1.68 | 3 | 0.84 | 3.70 |
| **Prairie dogs grassland** | h1 | 1.1 | 7 | 1.02 | 2 | 0.51 | 1.12 |
|  | h2 | 1.29 | 7 | 1.16 | 4.5 | 0.58 | 3.37 |
|  | h3 | 1.11 | 7 | 0.58 | 14 | 0.29 | 4.51 |
| **Prairie dogs grassland** | h1 | 1.57 | 7 | 0.9 | 0.6 | 0.45 | 0.42 |
|  | h2 | 1.52 | 7 | 1 | 2.6 | 0.50 | 1.98 |
|  | h3 | 1.49 | 8 | 1.14 | 3.9 | 0.57 | 3.31 |
|  | h4 | 1.55 | 7 | 4.04 | 13.1 | 2.02 | 41.02 |
| **Mesquite scrubland** | h1 | 1.56 | 8 | 1.28 | 2 | 0.64 | 2.00 |
|  | h2 | 0.94 | 7 | 1.08 | 3 | 0.54 | 1.52 |
|  | h3 | 1.14 | 7 | 0.6 | 5 | 0.30 | 1.71 |
| **Mesquite scrubland** | h1 | 1.3 | 7 | 2.08 | 1 | 1.04 | 1.35 |
|  | h2 | 1.04 | 7 | 1.06 | 6 | 0.53 | 3.31 |
|  | h3 | 0.97 | 7 | 0.44 | 13 | 0.22 | 2.77 |
| **Mesquite scrubland** | h1 | 1.34 | 7 | 0.58 | 3 | 0.29 | 1.17 |
|  | h2 | 1.58 | 7 | 1.02 | 3 | 0.51 | 2.42 |
|  | h3 | 1.53 | 7 | 0.86 | 4 | 0.43 | 2.63 |
|  | h4 | 1.29 | 8 | 1.96 | 10 | 0.98 | 12.64 |
